# The Effects of the Task Balance Training Program on the Glial Cell Line-Derived Neurotrophic Factor Levels, Cognitive Function, and Postural Balance in Old People

**DOI:** 10.1155/2022/9887985

**Published:** 2022-03-22

**Authors:** Meutiah Mutmainnah Abdullah, Andi Wardihan Sinrang, Djohan Aras, Jumraini Tammasse

**Affiliations:** ^1^Doctoral Programs, Faculty of Medicine, Hasanuddin University, Makassar, Indonesia; ^2^Department of Physiotherapy, Faculty of Nursing, Hasanuddin University, Makassar, Indonesia; ^3^Department of Physiology, Faculty of Medicine, Hasanuddin University, Makassar, Indonesia; ^4^Department of Neurology, Faculty of Medicine, Hasanuddin University, Makassar, Indonesia

## Abstract

Exercise in the form of physical activity can provide neuroprotective benefits. The purpose of this study is to determine the effect of the task balance training program (TBT program) on the glial cell-derived neurotrophic factor levels, cognitive function, and postural balance in old people. The population of this study was the old people members of the Batara Hati Mulia Gowa Foundation who were willing to participate in the study (*n* = 66). The sample of this study was obtained through a random sampling technique to determine the treatment (*n* = 32) and control (*n* = 34) groups. Before and after implementing the TBT program, glial cell-derived neurotrophic factor (GDNF) level measurement and cognitive function and postural balance assessment were performed. Cognitive function was measured by using Montreal cognitive assessment (MoCA). Postural balance was measured in two ways by using the timed up and go (TUG) test and Tinetti performance-oriented mobility assessment (POMA). The treatment group showed significantly greater changes than the control group in GDNF levels (2.24 (±0.63) vs. 1.24 (±0.43), *P* = 0.001), cognitive function (24.66 (±3.42) vs. 19.18 (±2.67), *P* = 0.001), and postural balance (TUG [14.00 (±4.04) vs. 18.68 (±3.98)]; POMA [26.53 (±1.74) vs. 23.47 (±3.06)], *P* = 0.001) after training. The treatment group also showed a significant relationship between GDNF levels and cognitive function (*r* = 0.840, *P* = 0.001) and postural balance (TUG [*r* = 0.814, *P* = 0.001]; POMA [*r* = 0.630, *P* = 0.001]). The TBT program affects the levels of GDNF in old people. The TBT program involves cognitive function improvement and affects postural balance changes in old people.

## 1. Introduction

Aging is a gradual process of the declining ability of tissues to repair or regrow themselves and maintain their normal functions. As the world's population rises, the population of older people is expected to continue to rise as well. The aging process causes continuous changes in old people's bodies including a gradual loss of function, weakness, illness, and even mortality. In old people, there is a decrease in muscle mass by 0.5%-1% per year, resulting in reduced strength and declining physical condition. Inactivity and the lack of exercise may exacerbate the physical condition of old people [1].

There are several types of degenerative diseases affecting the old people population, such as brain nerve damage or cognitive decline. A person is categorized as having a declining cognitive function, commonly known as dementia or senility, if s/he shows 3 or more of the following symptoms without disruption of consciousness: having attention and memory disturbances, place and time disorientation, and construction and execution inabilities (such as making decisions and solving problems). The cognitive function typically deteriorates as we age. As a result, this decline will impact the daily life of old people [2, 3].

Impairments in mobility and cognition are common in many neurological conditions, making each movement require more attention. Physiologically, attention is divided into two types: selective attention (focusing on one stimulus; for example, hearing the other person's voice when communicating in a noisy place) and divided attention (focusing on several stimuli; for example, calling while cooking). Divided attention is more often affected by neurological conditions. However, divided attention is necessary to perform two tasks simultaneously, such as walking and speaking [4, 5].

In everyday life, the ability to do work at the same time is necessary and expected. This represents the superiority of human evolution. However, when processing several stimuli and producing different responses, often there is a lower performance in one of the tasks being performed. Exercises are designed to improve walking and daily function in old people (e.g., people with Parkinson's). More and more rehabilitation strategies employ motor training, which facilitates patients by integrating functional activities to improve daily abilities. The task balance training (TBT) program is an advanced development of the strength and balance techniques and exercises with systematic procedures of repetitive muscle contractions. TBT can be classified into Single Task (ST) and Dual Task (DT). Some studies have proven that DT training was effective to improve balance and gait [1, 2]. It can reduce fall risk for elder people in a real life situation, and it also helps elder people to improve their walking speed [1, 3, 4]. Specifically, DT improves performance of episodic memory, sustained visual attention, functional mobility, cardiorespiratory fitness, lower limb strength resistance, agility, and quality of life [5].

Muscle adaptation in the loading process causes muscle hypertrophy as the result of the training. Exercise can also improve joint flexibility and range of motion and stimulate proprioception by increasing motor unit recruitment, which activates the Golgi tendon organs and muscle spindles. The more the number of muscle fibers innervated by a motor nerve, the greater the strength of the muscle. Throughout the training, the intrafusal and extrafusal threads receive sensory information, which will be relayed and processed by the brain to determine the necessary amount of force of the muscle cocontraction. Some of the responses will return to the extrafusal fibers and activate the Golgi tendon, resulting in better coordination between intrafusal and extrafusal fibers and afferent neurons in the muscle spindles and thus better proprioception. Sensory input, proprioception, nervous system, and muscular strength contribute to old people's balance [6–8].

Exercise is a physical activity that can provide neuroprotective benefits of increased neurogenesis, synaptogenesis, angiogenesis, and modulation of neurochemical levels in the brain. This process is mediated by a neurotrophic factor, glial cell-derived neurotrophic factor (GDNF), which is involved in neural plasticity, particularly in brain trauma and cognitive and memory impairment. Elevated GDNF levels can increase nerve cell survival in the nigrostriatal and other areas of the cerebral cortex. Activation of dopaminergic neurons produces neurotransmitters on the pathway to the basal ganglia to control body movements. Neurotransmitters are distributed in the prefrontal cortex which functions as memory storage and in the frontal lobe to maintain cognitive functions such as problem-solving, higher-order thinking, and learning [9–11].

GDNF is currently recognized as a critical component of nigrostriatal neuron development, maintenance, and protection and as a possible factor in the maintenance and repair of dopaminergic neurons damaged by Parkinson's disease. GDNF is a member of the neurotrophic factor family, a subfamily of the transforming growth factor-*β* (TGFb) superfamily, along with three other structurally similar factors—neurturin, artemin, and persephin. GDNF is also involved in the hippocampal synaptogenesis, playing a role as an instructional factor by activating presynaptic locations. The GDNF family receptor-1 (GFRa1) complex is needed for proper hippocampal circuit formation [11, 12]. There are few studies on the involvement of GDNF in cognitive function and postural balance in the elderly. The development of training methods is important to be observed that effect on molecular protein levels before and after the training. The results of this study are expected to provide a new scientific explanation regarding the TBT program in the dynamics of these neurotrophic factors. To the best of our knowledge, this study is the first attempt to investigate the benefits of the TBT program on changes in cognitive function and postural balance in the elderly through changes in GDNF levels. Based on these considerations, we hypothesize that the task balance training (TBT) program allows a significant improvement in cognitive function, postural balance, and levels of glial cell-derived neurotropic factor (GDNF) in the elderly. Therefore, this study is aimed at evaluating the effect of the TBT program on changes in cognitive function and postural balance with the Tinetti performance-oriented mobility assessment and the timed up and go (TUG) method in the elderly through changes in GDNF levels.

## 2. Materials and Methods

### 2.1. Participants

The population in this study was the old people members of the Batara Hati Mulia Gowa Foundation who were willing to participate in the study. Inclusion criteria are (a) those who have not had difficulty walking or fall within the last 3 months, (b) those who have no serious pain, musculoskeletal damage, or neurological damage, (c) those who understand the instructions of the examiner and can perform them, and they were between the ages of 60 and 75. Exclusion criteria are (a) a person with acute inflammation and (b) a person with visual and hearing problems. All participants understood the content of the study and participated voluntarily. The criteria for considering types of people included in studies should be sufficient to encompass the likely diversity and ensure the sample can carry out exercise without restriction of movement ability. The elderly have difficulty performing exercise movements such as walking if they have serious pain, muscle, and nerve disorders. The elderly with walking disorders, a history of falls, and muscle and nerve disorders can affect balance when they are given exercise; it can be limited to carrying out exercise movements such as walking exercises and standing exercises on one leg in a series of exercises. The random sampling approach was utilized to determine the participants of the treatment and control groups. The treatment group was assigned to the TBT program, while the control group was provided with therapeutic communication. It is an effective communication that is carried out consciously and designed in such a way by medical personnel to patients with the aim of helping patients to get positive health outcomes as expected [6]. In other words, it is a kind of communication that is aimed at developing the doctor-patient relationship, increasing patient satisfaction, and improving treatment adherence [7].

Blood sampling collection, Montreal cognitive assessment (MoCA), timed up and go (TUG) test, and Tinetti performance-oriented mobility assessment (POMA) were applied in both groups before and after intervention, respectively. This study received approval from the Health Research Ethics Commission, Faculty of Medicine, Hasanuddin University (828/UN4.6.5.31/PP36/2020). The research ethics recommendations included a requirement to provide an informed consent form and agreement sheet to participants who met the inclusion criteria. If the subject were willing to become a participant, they must sign the consent form. The sample who refused to participate would not be forced, and their rights would be respected. The second recommendation was anonymity, which required the author to give a specific code to each participant instead of listing participants' real names. Thirdly, the author must ensure confidentiality of the participants' information and only reported relevant information in the research results.

### 2.2. Intervention

The exercise program was performed 3 in three sessions a week for 4 weeks with 30 minutes per session. The exercise program comprised cognitive exercises and motoric exercises. Each exercise was performed for 30 sec followed by 30 sec of rest, and three sets were performed with 1 min break between the sets. The exercise intensity was 30-40% of the elderly's maximum heart rate range. The intensity complies with the American College of Sports Medicine guidelines for exercise testing and prescription when it comes to exercise recommendations for old people. This approach does have beneficial effects related to health, especially in the elderly. The details of the exercise program are shown in Table 1.

### 2.3. Data Collection

The instruments used to measure the variables studied consisted of a Riester mercury sphygmomanometer, OneMed stethoscope, questionnaire, informed consent, and stopwatch. The instruments for blood sampling collection consisted of a 3 cc syringe, 25 G wing needle, tourniquet, 3 cc EDTA tube, plaster, and alcohol cotton ball. The instruments for storing blood samples consisted of a centrifuge, Eppendorf tube, filter tip, micropipette, and -20°C refrigerators. To measure GDNF levels, the enzyme-linked immunosorbent assay (ELISA) test was performed using a GDNF kit. Blood tests were performed on all subjects after 12 h of fasting. The blood was drawn at the same time under the same conditions before and after the intervention. A disposable needle was used to draw 3 mL from the anterior cubital vein. The blood was stored in serum-separating tubes for serum and centrifuged for 10 min at 3,000 rpm; 200 *μ*L of the serum was stored in a sample tube. The GDNF levels were assessed using the enzyme-linked immunosorbent assay (ELISA) method with the GDNF ELISA kit (MyBioSource, Inc., San Diego, USA, MBS3800791), respectively. Cognitive function was measured by using Montreal cognitive assessment (MoCA). Postural balance was measured in two ways by using the timed up and go (TUG) test and Tinetti performance-oriented mobility assessment (POMA). The timed up and go (TUG) test was conducted to evaluate changes in dynamic balance following the intervention. The subject began in a seated position. At a start signal, they stood up, walked 3 m away, turned around, walked back, and returned to a seated position. The time was measured from the start signal to when the subject was seated in the chair again.

### 2.4. Statistical Analysis

The data were analyzed using univariate analysis (frequency distribution) to produce the distribution and percentage of each variable. Paired *t*-test was also conducted to determine the effect of the TBT program on GDNF levels, cognitive function, and postural balance. Paired sample *t*-test measured the difference experienced by the subjects from the same group after being subjected to treatment. Furthermore, to find the mean difference of each variable, an independent sample *t*-test was performed. The independent sample *t*-test was carried out to examine if there was a statistically significant mean difference between the two independent groups. To examine the correlation between GDNF levels with cognitive function and postural balance, Spearman's correlation test was performed using the SPSS program. The Spearman's correlation test was selected in this study because the type of data to be processed from both independent and dependent variables was ordinal data. Spearman's correlation test was also able to provide the correlation coefficient value and show the direction of the relationship, whether it was negative or positive.

## 3. Results

### 3.1. Sample Characteristics

The participant' characteristics are presented in Table 2. In the treatment group, the proportion of participants in the younger group (62.5%) is higher than that in the older group (37.5%). Similarly, in the control group, the proportion of participants in the younger group (76.5%) is also higher than that in the older group (23.5%). Age has a positive effect on pathophysiological changes, chronic disease, muscle weakness, and cognitive decline [8–11]. Because the physiological conditions and the ability of body functions in the elderly population also vary according to age, it is necessary to classify the elderly based on age. Several studies on the elderly so far have classified the elderly into a younger age group and an older age group [12–14]. The proportion of female participants in the treatment group (59.4%) is higher than that of male participants (40.6%). Likewise, the number of female participants in the control group (67.6%) is also higher compared to that of male participants (32.2%). In this study, both TUG and POMA tests are employed to examine the risk of falling in old people participants. The mean TUG test scores and POMA scores in the treatment and control group can be seen in Table 2.

### 3.2. The Effect of the TBT Program on GDNF Levels, Cognitive Function, and Postural Balance

Normality distribution tested with Shapiro-Wilk test on GDNF levels in the elderly obtained *P* > 0.05; thus, GDNF levels in the elderly obtained were normally distributed; cognitive function data distribution in the elderly obtained *P* > 0.05; thus, cognitive function in the elderly obtained was normally distributed, and balance data distribution in the elderly obtained *P* > 0.05; thus, the balance in the elderly obtained was normally distributed. A homogeneity test was carried out whose results showed that the significant value of Levene's test for equality of variances was more than 0.05, so it can be concluded that the data values of GDNF, MoCA, TUG, and POMA of the intervention group and the control group were homogeneous.

The mean GDNF level before treatment is 0.90 (±0.31). The GDNF level rises to 2.24 (±0.63) after treatment, suggesting a statistically significant difference in the mean GDNF levels before and after treatment (Table 2). The mean values of cognitive function pre- and posttreatment are 18.03 (±3.16) and 24.66 (±3.42), respectively; hence, there is a statistically significant difference between the mean value of cognitive function before and after treatment. The mean TUG test scores before and after treatment are 21.97 (±4.00) and 14.00 (±4.04), respectively. As a result, there is a statistically significant difference in the mean TUG test scores before and after treatment. The mean POMA scores pre- and posttreatment are 22.59 (±3.06) and 23.47 (±3.06), respectively. Thus, there is a statistically significant difference in the mean value of the POMA scores before and after treatment. The GDNF levels in the treatment group increased with a *P* value < 0.001, indicating that the TBT program influenced the GDNF levels. Cognitive function was significantly improved in the treatment group with a *P* value < 0.001, indicating that the TBT program affected cognitive performance. With a *P* value < 0.001 for both the TUG and POMA scores, it can be concluded that there is a significant improvement in postural balance in the treatment group, suggesting that the TBT affected postural balance (Table 3).

The mean values of GDNF levels for the treatment and control groups are 2.24 (±0.63) and 1.24 (±0.43), respectively. The mean values of cognitive function for the treatment and control groups are 24.66 (±3.42) and 19.18 (±2.67), respectively. The mean TUG scores for the treatment and control groups are 14.00 (±4.04) and 18.68 (±3.98), respectively. The mean POMA scores for the treatment and control groups are 26.53 (±1.74) and 23.47 (±3.06), respectively. Therefore, it can be concluded that there is a statistically significant difference in the mean POMA score between the treatment and control groups. All variables measured in this study showed a statistically significant difference between the treatment and control groups (*P* < 0.001) (Table 4). In other words, from those results, it can be seen that there is an increase in gait patterns and balance as measured by the Tinetti performance assessment and cognitive improvement as measured by the Montreal cognitive assessment.

**Figure 1:**
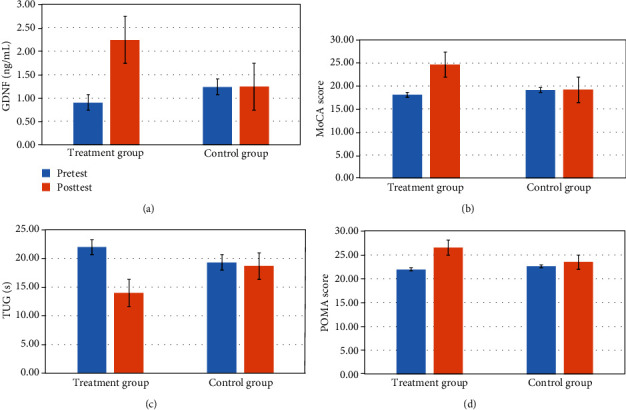
The comparison of GDNF levels, MoCA score, TUG score, and POMA score in the treatment group and the control group. (a) In pretest, GDNF in the treatment group was 0.90 ± 0.32 and that in the control group was 1.24 ± 0.42, and in the posttest, it was significantly higher in the treatment group (2.24 ± 0.63) than in the control group (1.24 ± 0.43) (*P* = 0.001). (b) In pretest, MoCA score in the treatment group was 18.03 ± 3.16 and that in the control group was 19.09 ± 2.71, and in the posttest, the treatment group (24.66 ± 3.42) was significantly higher than the control group (19.18 ± 2.67) (*P* = 0.001). (c) In pretest, TUG score in the treatment group was 21.97 ± 4.03 and that in the control group was 19.32 ± 3.98, and in posttest, it was significantly lower in the treatment group (14.00 ± 4.04) than in the control group (18.68 ± 3.98) (*P* = 0.001). (d) In pretest, POMA score in the treatment group was 21.91 ± 2.73 and that in the control group was 22.59 ± 3.09, and in posttest, it was significantly higher in the treatment group (26.53 ± 1.74) than in the control group (23.47 ± 3.06) (*P* = 0.001).

Comparison of the GDNF, MoCA score, TUG score, and POMA level score in pretest and posttest in the two groups revealed that the levels of the treatment group were not considerably different from those in the control group in the pretest. In the posttest, the levels of GDNF, MoCA score, and POMA score in the treatment group were considerably higher than those in the control group, and TUG score was lower in the treatment group, as shown in Figure 1.

The relationship between GDNF levels and cognitive function has a correlation coefficient value of 0.840. According to the degree of closeness between the two variables, there is a strong relationship and positive linear pattern, indicating that the higher one's GDNF level, the higher his/her cognitive function will be. The value of *P* < 0.001 signifies a relationship between GDNF levels and cognitive function. The correlation coefficient values of 0.818 (TUG) and 0.630 (POMA) indicate a correlation between GDNF levels and postural balance. According to the degree of closeness between the two variables, a strong relationship and positive linear pattern indicate that the higher one's GDNF levels, the better one's postural balance will be. The value of *P* < 0.001 signifies a relationship between GDNF levels and postural balance, either measured using the TUG test or POMA (Table 5).

## 4. Discussion

In old people, motor tasks require a higher level of control in executive information and memory processing, making it difficult for old people to perform activities simultaneously (e.g., walking while conversing). This indicates that one or both abilities have weakened due to declining metabolism in the frontal area. It is necessary to combine physical activity with cognitive stimulation; hence, a TBT program was designed to enhance cognitive capacities and slow brain aging by engaging cognitive and motor stimulations. Physical activity can provide neuroprotective benefits mediated by GDNF. GDNF is an essential protein that is required to develop, maintain, and protect nigrostriatal neurons, particularly in protecting and restoring dopamine neurons affected by Parkinson's. Therefore, it is necessary to determine whether the TBT program affects GDNF levels, cognitive function, and postural balance in old people. The effect of the TBT program on the GDNF levels, cognitive function, and postural balance in old people will be discussed according to the research objectives, as follows.

### 4.1. The Effect of the TBT Program on GDNF Levels in Old People

Glial cell line-derived neurotrophic factor (GDNF) supports neuroplasticity in the neuromuscular system from childhood to maturity. To maintain a healthy neuromuscular system, a steady supply of neurotrophic factors (NF) is required to support motor neuron growth and maturation. NFs contribute to the survival of motor neurons throughout their lives by encouraging their growth during embryonic development, maintaining them till adulthood, and regenerating them after injury [13]. In adults and old people, NF has been shown to act independently and synergistically in promoting healthy neuronal development and plasticity. Among the several known NFs, GDNF is the strongest neurotrophic factor in promoting motor neuron survival in vitro and in vivo [14]. GDNF has neurotrophic characteristics by being expressed in target skeletal muscle tissue, being transported retrogradely to the axonal cell body, and sustaining motor neurons throughout their lifespan. GDNF is up to 2,500 times more potent than other neurotrophins, preserving approximately 100% of axotomized motor neurons and the only factor capable of reversing axotomy-induced motor neuron atrophy [13].

The results of this study are in line with the study conducted by Gyorkos et al. [13] on rats being randomly assigned to running training (running group), swimming training (swimming group), or sedentary control group. The results indicated an increase in GDNF protein content (*P* < 0.05). GDNF is activity-dependent, and by changing the type and intensity of exercise, it is possible to increase the protein content of GDNF in slow and fast-twitch muscle fibers. This also strengthens the notion that GDNF affects the NMJ structure in slow and fast-twitch muscle fibers. Exercise may enhance the protein content of GDNF in skeletal muscle, resulting in improved NMJ plasticity and neuromuscular health [13]. Likewise, in the results of the study conducted by McCullough et al. [15], long-term exercise of voluntary running for 6 months showed a similar result (*P* < 0.05). These findings suggest a rise in the GDNF protein content in the muscles of the treated mice. It can be inferred that intensified physical activity increases structural neuroplasticity in the neuromuscular junction (NMJ) elements, resulting in improved neuromuscular function [15].

The training protocol was adequate to produce an increase in GDNF level, providing additional evidence for activity-dependent neurotrophic support mechanisms. The CSA of SOL muscle fibers reduced in response to this exercise plan shows that these muscles are engaged during this activity. The reduction in fiber diameter caused by training is an indication of recruitment. It can be viewed as an excellent adaptation to a faster oxygen, carbon dioxide, and waste product exchange, which prolongs fatigue time [13, 14].

### 4.2. The Effect of the TBT Program on Cognitive Function in Old People

A high level of cognitive function is critical for integrating and interpreting sensory-motor information necessary for maintaining equilibrium in the everyday environment. Individuals frequently complain about their memory as they age. However, compared to memory, other cognitive domains such as processing speed, executive function, and attention are more affected, which can have an impact on sensorimotor functions. Executive function and attention deficits have been linked to deteriorating physical function, such as slower gait and poor balance [5] (Taylor et al., 2019).

Exercise is a feasible strategy for improving cognition and delaying the onset of cognitive decline. This is a practical, nonpharmacological, and low-cost technique that has been thoroughly investigated: aerobic training, strength training, or a combination of both has been shown to improve brain structure and function, behavior, and cognition. Multimodal training is suggested in the recommendations. These recommendations are based on the studies that have investigated how exercise contributes to physical and cognitive function. It is advised for old people to perform a moderate exercise and monitor their heart rate while performing it [16].

The findings of this study are supported by the survey conducted by Jardim et al. [16], showing that there is an effect of Dual-Task Exercise (DTEx) on cognitive function in old people. The effects of GDNF on neuronal atrophy, a condition associated with cognitive decline in old age, were investigated in another study. The effects of GDNF on neuronal atrophy, a condition associated with cognitive decline in old age, were investigated in another study. The levels of GDNF on cognitive functions in the form of spatial learning and memory abilities showed significant changes in cognitive abilities in old age [17]. Decreased GDNF levels induce deregulation of the glutamate transporter, causing excitotoxicity in the nervous system resulting in dopaminergic degeneration [11]. The study investigated serum GDNF levels in aging that serum levels of GDNF are significantly decreased in older people. Age-related changes in innervation showed that innervation is regulated locally by neurotrophic factors exerting their activity at target sites [12, 13]. Cognitive function declines with age; the project also detects the lower levels of GDNF with impairment of attention, memory, and executive function in the elderly. GDNF has its neurotrophic effect on neurodegeneration where GDNF can exert neuroprotective and neurorestorative effects on substantia nigra neurons in the dopaminergic system [18]. GDNF can protect nigrostriatal dopamine neurons as a trophic and protective effect on neurons preventing neurons and glial cells from oxidative stress [10]. The more serious cognitive impairment with the lower levels of GDNF and four cognition-related neurotransmitters including dopamine metabolites homovanillic acid (HVA), acetylcholine (Ach), *γ*-aminobutyric acid (GABA), and 5-hydroxytryptamine (5-HT) [18].

Endogenous GDNF is necessary for cognitive abilities, as shown in a study of heterozygous mice with targeted GDNF gene deletion which resulted in a reduced spatial learning capacity [19]. In the present study based on the results of the analysis, it was found that in the treatment group seen from the results of the MoCA measurement there was a significant increase in cognitive function that occurred in our subjects performing the TBT program, while in the control group there was no significant change. This mode of exercise contains task of cognitive component performed in conditions of more complexity of exercise simultaneously balance and cognitive [16]. Linear cognitive improvement has a significant increase in GDNF in the treatment group and not significant in the control group. The elderly with low GDNF levels had spatial memory and learning impairment about time and place orientation, repeating words, sentences, as well as the ability to recall and rerecognize locations and events that have passed in everyday life. GDNF facilitates hippocampal neural progenitors and induces neurogenesis in the dentate gyrus. GDNF-mediated hippocampal neurogenesis contributed to the rescue of the cognitive impairment, an enhanced functionality of the antioxidant mechanisms protecting from the oxidative stress also against cognitive deficit [17]. Multiple pathways of GDNF neuroprotection are in involved GDNF signaling and survival pathways [12]. GDNF induces the activity of the antioxidant enzymes glutathione peroxidase, superoxide dismutase, and catalase in neurons [15]. The oxidative damage derives from overaccumulation of iron, mitochondrial dysfunction, and other interacting mechanisms that generate ROS in the vulnerable neuronal population [20].

There is mounting evidence that GDNF levels may play a role in the etiology and progression of illness, as they do in Alzheimer's disease with cognitive impairment. GDNF levels were shown to be strongly associated with cognitive impairment (*P* < 0.001) [17]. The study discovered that GDNF levels fell considerably in people with cognitive impairment, implying that GDNF may have a role as a disease biomarker. This finding is consistent with Wang et al. [18], who attempted to determine whether GDNF levels in plasma were abnormal in late-onset depression (LOD) and whether they were linked with cognitive impairment in LOD [18]. GDNF production is believed to play an active role in neuronal survival and plasticity. GDNF-mediated hippocampal neurogenesis contributed to the rescue of the cognitive impairment, an enhanced functionality of the antioxidant mechanisms protecting from the oxidative stress also against cognitive deficit [17]. GDNF induces increased neurotransmitter function, as previously demonstrated by increased synthesis of the neurotransmitters acetylcholine, dopamine, and serotonin [12, 18]. GDNF facilitates hippocampal neural progenitors and induces neurogenesis in the dentate gyrus [19]. The increase in GDNF in aged hippocampus showed an increase in learning and memory functions, while the decreased GDNF showed cognitive impairment. Induction of strong upregulation protects neurons from atrophy and degeneration [20, 21].

### 4.3. The Effect of the TBT Program on Postural Balance in Old People

Walking and falling disorders are problems that often occur in old people patients. In old people, there is a physiological decline in the musculoskeletal system, which manifests in a reduction of the number and size of muscle fibers, resulting in a weaker lower extremity muscle, endurance, coordination, and limited range of motion (ROM). Balance is a complex positional defense against outside interference. Impaired balance and gait and weakness of lower extremity muscles can cause falls in old people [22, 23].

Falls are the main cause of morbidity in old people patients. The TUG test is a sensitive and objective method of assessing balance and walking disorders. TUG test evaluated the time taken to complete the entire series of tests. Tinetti POMA, on the other hand, can provide additional benefit as the assessment minimize falls and at the same time train physical balance in old people. Physical balance exercise is an exercise to control movement and position in the center of the body. This exercise is also an essential component in maximizing other physical activities [23].

Physical activity appears to help prevent premature death of dopamine cells. In the basal ganglia, exercise-induced improvement in the synthesis of GDNF has been observed. In parkinsonism mice (or) monkeys, motor deterioration can be delayed and dopamine levels in the brain can be preserved by running on a treadmill [20, 24].

The results of this study are in line with the study conducted by Marques et al. [25], which showed that there is a relationship between balance training and the frequency of falls in old people. The frequency of falls in the group that has been given training is lower than that in the control group which falls once in the experimental group was 3.13%, while in the control group was 8.82% and falls 2 times in the experimental group was none, while in the control group was 5.88%. The frequency of falls with the elderly falls more often due to age-related strength deficits and impaired stability control [23]. Falls occurred most frequently while walking during the daytime and in the room. Exercise provides benefits for both factors that experience a decrease (balance and strength) can be trained at the same time [16]. Management of balance disorders is an approach used in fall prevention interventions [25]. Similarly, in the study by Alfieri et al. [26], it was found that balance training affected the functional mobility and harmony of senior participants in the treatment group. The frequency of falls decreased along with the increase in body balance function in the elderly after the intervention. Changes in the risk of falling and falling elderly people (faller) are likely to be a consequence of changes in balance and strength seen to increase after the intervention program concerning improvements in balance and functional gait performance [6, 26]. The number of falls is reduced during exercise in the elderly. Continuous training is necessary to maintain the benefits of exercise against risk factors for falls [22, 23].

In conclusion, the task balance training program improved GDNF, cognitive function, and postural balance in old people. Therefore, it could be suggested as an intervention program for improving the cognitive function and postural balance in old people. The limitations of this study were a relatively small sample size, the intervention period was short, and no laboratory examination of neurotrophic factor receptors which specifically bind to GDNF that might affect. Further study should be conducted to analyze the role of GDNF receptors through exercise on cognitive function and postural balance in old people.

## Figures and Tables

**Table 1:** Task balance training program.

Exercise stage	Program	Time	Week
Warm-up	Stretching	5 min	
Main exercise	(i) Walking straight simultaneously by answering questions about day, date, month, year, person, and certain places (ii) Walking sideways by mentioning names of objects and words starting with certain letters (iii) Walking straight (tandem gait) heel position touch the toes of the other while counting numbers (iv) Walking past the obstacle by repeating words and sentences	30 sec exercise; 30 sec rest/set 3 sets 20 min	1-4
Cooldown	Stretching	5 min	

The time of a single round of exercise was set at 30 min, which included the warm-up (5 min), main exercise (20 min), and cooldown (5 min). Each exercise was performed for 30 sec followed by 30 sec of rest, and three sets were performed with 1 min break between the sets.

**Table 2:** Participants' characteristics.

Baseline characteristics	Value	
	Treatment (*n* = 32)	Control (*n* = 34)
Age (years old)	68.09 (±3.00)	67.32 (±2.28)
Younger old people (60-69)	20 (62.5)	26 (76.5)
Older old people (≥70)	12 (37.5)	8 (23.5)
Sex		
Male	13 (40.6)	11 (32.4)
Female	19 (59.4)	23 (67.6)
GDNF level (ng/mL)	0.90 (±0.31)	1.24 (±0.42)
Cognitive function (MoCA)	18.03 (±3.16)	19.09 (±2.71)
Postural balance (TUG)	21.97 (±4.03)	19.32 (±3.98)
Postural balance (POMA)	21.91 (±2.73)	22.59 (±3.09)

Values are presented as number (%) or mean ± standard deviation. GDNF: glial cell line-derived neurotrophic factor; MoCA: Montreal cognitive assessment; TUG: timed up and go; POMA: performance-oriented mobility assessment.

**Table 3:** Comparison of GDNF levels, cognitive function, postural balance (TUG), and postural balance (POMA) between before and after intervention.

Variable	Group	Pre	Post	*P* value
GDNF levels (ng/mL)	Treatment	0.90 ± 0.32	2.24 ± 0.63	0.001
	Control	1.24 ± 0.42	1.24 ± 0.43	0.815
Cognitive function (MoCA)	Treatment	18.03 ± 3.16	24.66 ± 3.42	0.001
	Control	19.09 ± 2.71	19.18 ± 2.67	0.697
Postural balance (TUG)	Treatment	21.97 ± 4.03	14.00 ± 4.04	0.001
	Control	19.32 ± 3.98	18.68 ± 3.98	0.034
Postural balance (POMA)	Treatment	21.91 ± 2.73	26.53 ± 1.74	0.001
	Control	22.59 ± 3.09	23.47 ± 3.06	0.001

Values are presented as mean ± standard deviation. GDNF: glial cell line-derived neurotrophic factor; MoCA: Montreal cognitive assessment; TUG: timed up and go; POMA: performance-oriented mobility assessment.

**Table 4:** Comparison of GDNF levels, cognitive function, postural balance (TUG), and postural balance (POMA) between the treatment and control groups.

Variable	Treatment	Control	*P* value
GDNF (ng/mL)	2.24 ± 0.63	1.24 ± 0.43	0.001
Cognitive function (MoCA)	24.66 ± 3.42	19.18 ± 2.67	0.001
Postural balance (TUG)	14.00 ± 4.04	18.68 ± 3.98	0.001
Postural balance (POMA)	26.53 ± 1.74	23.47 ± 3.06	0.001

Values are presented as mean ± standard deviation. GDNF: glial cell line-derived neurotrophic factor; MoCA: Montreal cognitive assessment; TUG: timed up and go; POMA: performance-oriented mobility assessment.

**Table 5:** Relationship between GDNF levels, cognitive function, and postural balance.

Variable	Correlation coefficient	*P* value
GDNF levels-cognitive function (MoCA)	0.840	0.001
GDNF levels-postural balance (TUG)	0.818	0.001
GDNF levels-postural balance (POMA)	0.630	0.001

GDNF: glial cell line-derived neurotrophic factor; MoCA: Montreal cognitive assessment; TUG: timed up and go; POMA: performance-oriented mobility assessment.

## Data Availability

The datasets generated and analyzed during the present study are available from the corresponding author on reasonable request.
